# A Use of Tritium-Labeled Peat Fulvic Acids and Polyphenolic Derivatives for Designing Pharmacokinetic Experiments on Mice

**DOI:** 10.3390/biomedicines9121787

**Published:** 2021-11-29

**Authors:** Gennady A. Badun, Maria G. Chernysheva, Yury V. Zhernov, Alina S. Poroshina, Valery V. Smirnov, Sergey E. Pigarev, Tatiana A. Mikhnevich, Dmitry S. Volkov, Irina V. Perminova, Elena I. Fedoros

**Affiliations:** 1 Department of Chemistry, Lomonosov Moscow State University, 119991 Moscow, Russia; badunga@yandex.ru (G.A.B.); masha.chernysheva@gmail.com (M.G.C.); zhernov@list.ru (Y.V.Z.); tanya.4erkasova8q@gmail.com (T.A.M.); dmsvolkov@gmail.com (D.S.V.); 2 Department of General Hygiene, F. Erismann Institute of Public Health, I.M. Sechenov First Moscow State Medical University (Sechenov University), 119435 Moscow, Russia; vall@mail.mipt.ru; 3 National Research Center, Institute of Immunology FMBA of Russia, 115522 Moscow, Russia; alinaporoshina145@gmail.com; 4 N.N. Petrov National Medical Research Center of Oncology, Ministry of Health of Russian Federation, 197758 Saint Petersburg, Russia; spigarev@gmail.com (S.E.P.); elenafedoros@gmail.com (E.I.F.); 5 Nobel Ltd., 192012 Saint Petersburg, Russia; 6 V.V. Dokuchaev Soil Science Institute of RAAS, 119017 Moscow, Russia

**Keywords:** fulvic acid, lignin, polyphenolic composition, tissue distribution, pharmacokinetics, intravenous, oral, tritium label

## Abstract

Natural products (e.g., polyphenols) have been used as biologically active compounds for centuries. Still, the mechanisms of biological activity of these multicomponent systems are poorly understood due to a lack of appropriate experimental techniques. The method of tritium thermal bombardment allows for non-selective labeling and tracking of all components of complex natural systems. In this study, we applied it to label two well-characterized polyphenolic compounds, peat fulvic acid (FA-Vi18) and oxidized lignin derivative (BP-Cx-1), of predominantly hydrophilic and hydrophobic character, respectively. The identity of the labeled samples was confirmed using size exclusion chromatography. Using ultra-high resolution Fourier transform ion cyclotron resonance mass spectrometry (FT ICR MS), key differences in the molecular composition of BP-Cx-1 and FA-Vi18 were revealed. The labeled samples ([^3^H]-FA-Vi18 (10 mg/kg) and [^3^H]-BP-Cx-1 (100 mg/kg)) were administered to female BALB/c mice intravenously (i.v.) and orally. The label distribution was assessed in blood, liver, kidneys, brain, spleen, thymus, ovaries, and heart using liquid scintillation counting. Tritium label was found in all organs studied at different concentrations. For the fulvic acid sample, the largest accumulation was observed in the kidney (C_max_ 28.5 mg/kg and 5.6 mg/kg, respectively) for both routes. The organs of preferential accumulation of the lignin derivative were the liver (C_max_ accounted for 396.7 and 16.13 mg/kg for i.v. and p.o. routes, respectively) and kidney (C_max_ accounted for 343.3 and 17.73 mg/kg for i.v. and p.o. routes, respectively). Our results demonstrate that using the tritium labeling technique enabled successful pharmacokinetic studies on polyphenolic drugs with very different molecular compositions. It proved to be efficient for tissue distribution studies. It was also shown that the dosage of the polyphenolic drug might be lower than 10 mg/kg due to the sensitivity of the ^3^H detection technique.

## 1. Introduction

Natural medicines have been used for ages in Asia to prevent and treat diseases, and in recent time, natural products garnered substantial appreciation in the West [1]. The main reason is their beneficial safety profile characterized by low toxicity and rare adverse effects. At the same time, well-documented studies on the safety and efficacy of natural products are very scarce. These parameters depend heavily on both the composition and pharmacokinetics of components comprising these complex systems. Hence, there is an urgent need to develop a pharmacological basis for estimation of the efficacy of natural medicine products [2]. Of particular interest is bioavailability, which defines the rate and extent of absorption of an active ingredient of an administered drug. Depending on the administered dose, plant polyphenols are reported to exert protective or toxic action associated with their antioxidant or pro-oxidant effects [3,4]. A better understanding of pharmacokinetics and bioavailability of phytopharmaceuticals is a prerequisite for establishing rational dosage regimens. In addition, data on absorption and distribution of active ingredients in body fluids and tissues are a requirement in drug development, regardless of the route of administration [5].

Natural products of polyphenolic nature are diverse and include plant polyphenols (e.g., flavonoids, tannins), lignins, and humic substances (HS). Oxidized lignin derivatives with antioxidant activity can serve as a promising source of natural polyphenolic additives, which could successfully replace synthetic and semisynthetic compounds used in cosmetic, pharmaceutical, and polymer compositions [6,7]. BP-Cx-1 is a water-soluble multicomponent composition of polyphenolic compounds derived from lignin [8]. BP-Cx-1 is used as a platform chemical for a number of compositions with different activities. For example, BP-C3 is a formulation of BP-Cx-1 with iron complex, selenium, ascorbic acid, and retinol. Long-term treatment with BP-C3 causes anticarcinogenic and geroprotective activity in female SHR mice [9]. BP-C3 was found to reduce the toxic effect of 5-fluorouracil on hematopoiesis and intestinal epithelium, evidenced by preserved organ/body ratios for the lymphopoietic organs, reduced anemia, and survival of the intestinal crypts [10]. BP-C2 is a composition of BP-Cx-1 and ammonium molybdate, which acts as a radioprotector/radiomitigator [11].

HS possess antioxidant activity due to the presence of multiple phenolic groups [12,13,14,15]. They are also known to display antiviral activity, anti-inflammatory, and other types of biological activity [16,17]. The prospects of fulvic acid use as a pharmaceutical excipient are discussed explicitly in several publications by Mirza et al. [18,19]. Other authors suggest using peat humic acids for different therapeutic applications [20]. Despite these multiple reports on the prospects of medicinal applications of HS, the data on their pharmacokinetics are very scarce, as shown in the recent reviews and references therein [21,22]. This is mostly due to complexity of these natural mixtures.

Various methods were developed for studying pharmacokinetics of complex mixtures. High-pressure liquid chromatography mass spectrometry techniques (HPLC MS) can be used for specific and sensitive detection of individual active compounds in blood plasma, taking into account their stereochemistry [23]. Different modifications of HPLC MS allow for simultaneous detection of up to ten individual components of plant extracts in biological samples [24,25]. Still, a lack of single fragmentation pattern and of a library of standard compounds prevent the broader use of these methods for studying the pharmacokinetics of complex mixtures. The most powerful analytical technique for non-target analysis of natural complex mixtures is Fourier transform ion cyclotron resonance mass spectrometry (FT ICR MS) [26,27,28]. It is characterized by an unprecedented resolving power reaching 1,600,000 at *m/z* = 400 for a flagman 21 T instrument and root mean square mass measurement accuracy below 100 ppb [29]. Its application in the analysis of HS [30,31] and lignins [32,33] has provided for substantial advances in understanding the structures of the molecular components that comprise these complex mixtures. This has enabled dereplication studies of HS aimed at examining feasible structures responsible for biological activity of HS, e.g., antiviral activity [34]. However, severe limitations of FT ICR MS in identification of individual components of complex mixtures due to isomeric diversity [35] hinders its application for pharmacokinetics studies.

From this point of view, tritium thermal bombardment, which enables incorporation of the tritium label into carbon backbone by exchanging non-labile H-atoms with ^3^H-atoms, is a promising technique for non-selective labeling of natural multicomponent systems. The use of this technique for HS was described in our previous publications [36]. Of particular importance is that this technique ensures homogenous distribution of the [^3^H] label among all components of HS. These [^3^H]-labeled HS samples were successfully used for estimating the uptake of HS by bacterial cells (*Escherichia coli*) [37] as well as for visualizing HS distribution in the plant tissues (wheat plants) [38].

This study is devoted to estimating the suitability of tritium-labeled natural products for use in pharmacokinetic studies. We incorporated the ^3^H label into very different polyphenolic materials, oxidized lignin derivative and peat fulvic acid, to reach this goal. They were further administered in mice using intravenous injection and gavage. Substantially different doses were administered to determine the range of concentrations that can be reliably detected with a use of liquid scintillation technique in the internal organs of mice.

## 2. Materials and Methods

### 2.1. Sources and Structural Characterization of the Humic and Lignin-Derived Materials

Humintech Ltd. (Grevenbroich, Germany) provided a sample of FulvAgra^®^ fulvic acid (FA-Vi18). The sample is an ion-exchange resin isolate from the underground water leaching the peat deposit. Nobel Ltd. (St. Petersburg, Russia) provided a sample of the lignin-derivative (BP-Cx-1).

Elemental analyses (C, H, N) were performed on a Vario El Cube elemental analyzer. Ash content was determined manually. Oxygen content was calculated as a difference between the total weight (100%) and the sum of elements (CHN, % mass) calculated on an ash-free basis. The H/C and O/C atomic ratios were derived from the contents of the elements calculated on an ash-free basis (Table 1).

The quantitative solution-state ^13^C NMR spectra were acquired as described in our previous studies [39] using a Bruker Avance 400 MHz NMR spectrometer (Bruker BioSpin Ltd., Rheinstetten, Germany). The spectrometer operates at 100 MHz ^13^C frequency and is located at the Lomonosov MSU, Moscow, Russia. Inverse gated decoupling and relaxation delay of 8 s were used to provide quantitative conditions. The FA sample was dissolved in 0.1 M NaOD at a concentration of 100 g/L. The assignments were made after [39] and were as follows (in ppm): 5–45—aliphatic H and C-substituted C atoms (CH_n_), 45–56—aliphatic C atoms in methoxyl groups (CH_3_O), 56–111—aliphatic O-substituted C atoms (C_alk_O), 111–144—aromatic H and C-substituted atoms (C_ar_H(C)), 144–167—aromatic O-substituted C-atoms (C_ar_O), 167–188—C atoms of carboxylic and esteric groups (COO), 188–220—C atoms of quinonic and ketonic groups (C=O).

SEC was performed according to [40] using Toyopearl HW-55S resin (Tosoh Bioscience, Griesheim, Garmany) as column packing. Column dimensions were 2 cm × 25 cm. A UV absorbance detector was used at the wavelength of 254 nm. Phosphate buffer (0.028 M) at pH 6.8 was used for elution. The elution rate was 1 mL/min. Polystyrenesulfonates were used for calibration. Molecular weight in peak (*M*p) was used as an estimate of molecular weight.

Ultra-high-resolution mass spectra were acquired on a Bruker solariX 15 T FT ICR mass spectrometer (Bruker Daltonics, Bremen, Germany) equipped with a 15 Tesla superconducting magnet and an Apollo II source in negative electrospray ionization mode according to standard conditions [41]. Samples were injected with a constant flow rate of 120 µL/h, nebulizer gas pressure of 2.2 bar, and drying gas pressure of 4 bar at 200 °C. Accumulation time was 0.4 s. The applied ESI voltages were 3600 V capillary voltage and −500 V end plate offset. The spectra were acquired using a time transient of 4 MW. MS parameters were optimized to reach a maximum of sensitivity in the *m/z* range 150–1000. Transfer optic parameters were ToF of 0.6 ms, frequency of 4 MHz, and RF amplitude of 175 V. In total, 300 scans were acquired for each sample.

Molecular assignments were made using lab-made Transhumus software based on the open-source R environment designed by A. Grigoryev and plotted into van Krevelen diagrams. The parameters used for formula assignments were set with sensible chemical constraints according to the literature: O/C ratio below or equal to 1, H/C between 0.3 and 2.2, element counts (C atoms up to 120, H atoms up to 200, oxygen atoms between 1 and 60, N atoms up to 1), and a mass accuracy window below 0.5 ppm (Da/MDa) [42]. The van Krevelen diagram was binned into 20 cells, which were assigned to seven chemotypes: condensed tannins, phenylisopropanoids, terpenoids, lipids, proteins, carbohydrates, and hydrolyzed tannins. Occupational densities on van Krevelen diagrams for each chemotype (total intensity of all signals corresponding to certain cells) were calculated after Perminova and the resulting values were displayed on the histogram [43]. Common and unique molecular formulas have been identified among the assigned formulas for BP-Cx-1 and FA-Vi18.

### 2.2. Tritium Labeling of the Fulvic Acid and the Lignin Derivative Used in This Study

The tritium labeling of BP-Cx-1 and FA-Vi18 samples was carried out as described in [36,44]. The method implies substitution of hydrogen with tritium in the C-H bonds of chemical compounds. It is achieved by explosion of the labeled compounds to a flux of “hot” tritium atoms obtained by heating up a tungsten wire using electric current. To minimize side processes, labeling is carried out by bombardment of a thin frozen layer of the target on the walls of a reaction vessel, which is obtained by lyophilization of an aqueous solution. As a result, the non-selective substitution of hydrogen with tritium occurs while maintaining the chemical composition and structure of the molecules. Exchangeable tritium atoms were removed with dialysis against 0.028 M phosphate buffer, as shown in Supplementary Figure S1. Characteristics of the labeled compounds were confirmed using SEC according to the procedure described in [44,45].

Using the parent sample, the concentration of [^3^H]-FA-Vi18 was adjusted to 0.93 mg/mL (0.093%) before administration to mice in the dose of 10 mg/kg (based on the administration volume of 0.2 mL per animal with a bodyweight of 18.6 g). The resulting specific activity of [^3^H]-FA-Vi18 was 139 μCi/mg. The sample of [^3^H]-BP-Cx-1 was dissolved in 0.9% NaCl and pH was adjusted to 7.0 with 10 mM Na_2_HPO_4_. Using the parent sample, concentration of [^3^H]-Bp-Cx-1 was adjusted to 1% before administration to mice in the dose of 100 mg/kg (based on the administration volume of 0.2 mL per animal with the bodyweight of 20 g). The resulting specific activity of [^3^H]-BP-Cx-1 was 14.2 μCi/mg.

### 2.3. Animals Welfare

Female BALB/c mice (animal facility Stolbovaya, Moscow District, Russia) were quarantined for 14 days upon delivery. The animals were kept in T2 type IVC cages under artificial 12 h light/dark cycle conditions, 21 ± 2 °C, average humidity of 20–50%, and ad libitum access to laboratory chow (Laboratorkorm LLC, Moscow, Russia) and tap water.

Experimental animals were handled under the Guide for the Care and Use of Laboratory Animals, 8th edition. The study protocol was approved by the Local Ethics Committee of the N.N. Petrov National Medical Research Center of Oncology (protocol no. 8; dated 18 June 2020). Experimental animals found in a moribund condition and at the end of observation were euthanized with the guillotining method.

### 2.4. Animal Study Design

Mice were administered the prepared tritium-labeled samples once via injection into the tail vein using an insulin syringe with a 27 G needle or by gavage utilizing an insulin syringe and a metal probe with a Luer type connector. The intravenous administration was selected for achieving maximum bioavailability. Administration by gavage was used to mimic the most preferable oral use of the drugs. Administration volume was 0.2 mL per mice. Intravenous administration was carried out using a plastic restrainer without anesthesia.

Female BALB/c mice (72 animals) with body weights ranging from 18.5 to 22.5 g were divided into groups, 3 per each sampling time point. For the i.v. route, sampling was performed 5 min, 30 min, 1 h, 2 h, 6 h, and 12 h (24 h for [^3^H]- FA-Vi18) after injection. For the oral route, sampling was performed 30 min, 60 min, 2 h, 6 h, 24 h, and 48 h after administration. The mice were euthanized at the appropriate time point by the guillotining method to ensure the fastest possible blood circulation stop and blood collection. For both routes of administration, a whole blood sample was taken into a vial with K_2_EDTA. Then, internal organs were excised to further evaluate the content of the labeled compound in them (brain, liver, kidneys (both), heart, spleen, thymus, ovaries (both)). The organs thoroughly cleaned of surrounding tissues were washed from blood in a cold solution of 0.9% sodium chloride (400 mL, the solution was changed after every 3 animals at the one-time point). Then, fluid residues were removed using filter paper, and organs were placed in pre-marked and weighted glass vials for further detection of tritium.

### 2.5. Liquid Scintillation Counting (LSC)

The vials with organs were weighed, the weight of organs was calculated, and the organs were dissolved in a solvent Solvable (PerkinElmer, Inc., Waltham, MA, USA) according to the procedure recommended by the manufacturer. Then, all or part of the preparation was mixed with a scintillation cocktail UlimaGold (PerkinElmer, Inc., Waltham, MA, USA), and tritium radioactivity was measured using a liquid scintillation spectrometer Tri-Carb 1600 (PerkinElmer, Inc., Waltham, MA, USA).

The content of the drug in the sample C*o* (mg/kg) was calculated with the equation:

(1) $$Co=\frac{DPM-BG}{P*A*W*2220000},$$

where *DPM* is the measured radioactivity (disintegrations per minute); *BG* is the background value, which was from 100 to 200 dpm for different series of preparations; *P* is the part of the sample taken for the measurement; *A* is the specific radioactivity of the [^3^H]-FA-Vi18 or [^3^H]-BP-Cx-1 (μCi/mg); *W* is the weight of the organ or tissue sample (10^−3^ kg); and 2,220,000 is the conversion factor from dpm to μCi.

### 2.6. Calculation of Pharmacokinetic Parameters

The calculated individual values from three animals in the group were averaged (Supplementary Tables S1 and S2); the arithmetic means were used to assess the bioavailability and tissue distribution profile of the two compositions and to calculate the main pharmacokinetic parameters. Pharmacokinetic calculations were performed using the R Program (version R-3.6.2 for Windows, 2019-12-12) (R Foundation for Statistical Computing, Vienna, Austria) [46].

The following pharmacokinetic parameters were calculated: C_max_ is the highest concentration of the sample; AUC_0-t_ is the area under the concentration curve from the time 0 to the last determined concentration at a time point t; Lz is the elimination rate constant; AUC_0-inf_ is the area under the curve from time 0 extrapolated to infinite time; T_1/2_ is the half-life; and MRT is the mean retention time in the body.

## 3. Results

### 3.1. Structural Characteristics of the Parent and ^3^H-Labelled Samples of Fulvic Acid and Lignin Derivative Used in this Study

The samples of peat FA and lignin derivative were characterized for elemental composition, structural group composition, and molecular weight distribution. The obtained values of H/C and O/C atomic ratios for the FA sample used in this study (Table 1) are indicative of the predominantly aliphatic character of this sample (H/C is 1.27, which is much higher than one). The high O/C ratio (0.68) is, in turn, indicative of the high contribution of oxidized groups into the structure of this sample. These data are in sync with those published for FA in the literature [47] and with the direct measurements of structural group composition of this sample performed with ^13^C NMR spectroscopy (Table 2).

Unlike FA-Vi18, the sample of water-soluble lignin derivative BP-Cx-1 was characterized by a low oxidation degree (O/C ratio of 0.35) and high contribution of aromatic fragments into molecular structures (H/C ratio of 0.75) (Table 1).

The structural group composition is presented in Table 2. The FA-Vi18 sample is characterized by the relatively low contribution of aromatic carbon (less than 30% of the total carbon). At the same time, it contained a factor of 1.5 larger amount of aliphatics as compared to aromatic carbon, which explains its low lipophilicity index (0.67; ΣCar/ΣCalk) [48] and indicates high hydrophilicity of the FA sample. This sample is also characterized by high contribution of carboxyl and carbonyl groups, >25% of the total C, which corroborates well with high oxidation degree of the sample as measured by the elemental analysis. It is typical for fulvic acids.

A distinctive feature of the lignin derivative sample (BP-Cx-1) is the presence of a sharp peak at 55–58 ppm on the ^13^C NMR spectrum (Supplementary Figure S1), which is related to methoxy groups—constitutive parts of lignin. High values of spectral density in the range from 108 to 165 ppm of ^13^C NMR are consistent with the predominantly aromatic nature of this compound. The aromatic carbon content in this sample accounts for 47% of the total C: it represents the main part of BP-Cx-1. This sample contains carboxyl and carbonyl groups formed during the oxidation of the starting lignin material. These two groups constitute 17%, which is significantly less than in the FA-Vi18 sample (25%); these data are in agreement with the lower oxidation of BP-Cx-1. The lipophilicity index of BP-Cx-1 is 1.3, which is two times larger than that of the FA-Vi18 sample. The obtained values indicate the high hydrophobicity of the BP-Cx-1 sample. In general, the structural group composition of the studied compound agrees well with the data for the lignin starting material—a cross-linked polymer composed of phenylpropanoid units [49].

**Figure 1:**
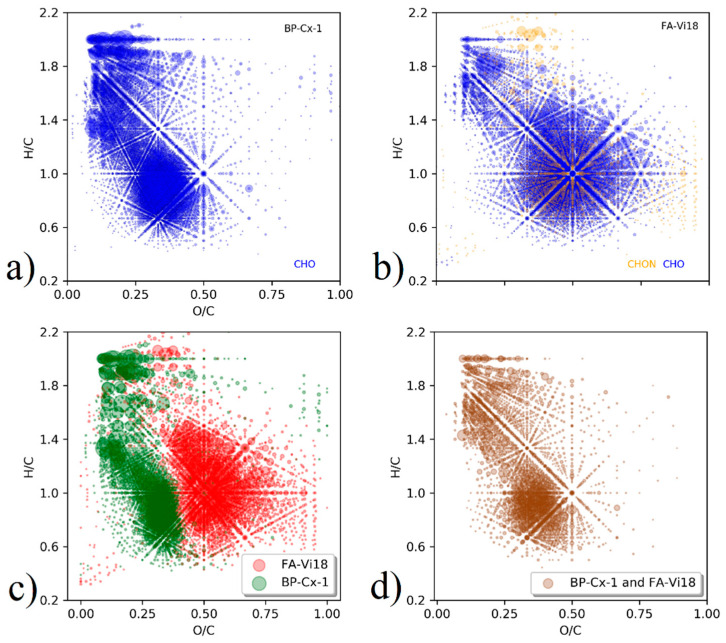
Van Krevelen diagrams of the (**a**) BP-Cx-1, (**b**) FA-Vi18, (**c**) unique formulae for BP-Cx-1 (green) and for FA-Vi18 (red), and (**d**) common formulas both BP-Cx-1 and FA-Vi18 (brown). The dot size corresponds to the peak intensity in the mass list. On van Krevelen diagrams of all assigned formulas (**a**,**b**), CHO formulae are highlighted in blue, and CHON in yellow.

The molecular compositions of BP-Cx-1 and FA-Vi18 samples were analyzed using FT ICR MS. Van Krevelen diagrams of assigned molecular formulas for BP-Cx-1 and FA-Vi18 are shown in Figure 1a,b.

The most substantial differences between BP-Cx-1 and FA-Vi18 samples were observed in the high content of low-oxidized species (O/C < 0.5) in the lignin derivative, and in predominance of highly oxidized components (O/C > 0.5) in the sample of fulvic acids. Thus, the lignin-derivative was characterized with the dominance of condensed aromatic compounds and lignin-like species, which occupy the range with the same O/C values (O/C < 0.5) and different H/C values (H/C < 1.0 and 1.0 < H./C < 1.4), respectively. This is consistent with hydrophobic character of the lignin derivative under study.

Despite the substantial differences between the molecular compositions of the samples used in this study, they shared 2881 common formulae out of total 6382 and 6487 formulae assigned to FT ICR MS data on BP-Cx-1 and FA-Vi18, respectively. Van Krevelen diagrams for the unique and common molecular components of BP-Cx-1 and FA-Vi18 are shown in Figure 1c,d. It can be seen that the both samples shared the common molecular compositions in the range of terpenoic, lignin-like, and flavonoid structures (Figure 1d). The molecular formulae unique to BP-Cx-1 mainly populate the region of condensed tannins (O/C < 0.5, H/C < 1.0) and phenylisopropanoids (O/C < 0.5, 1.0 < H/C < 1.4). The molecular components that are unique to FA-Vi18 were completely absent in these regions of the van Krevelen diagram. On the contrary, the unique molecular components of FA-Vi18 were mainly represented by hydrolyzed-tannins-like formulae (O/C > 0.5, 1.0 < H/C < 1.4), which were absent in the BP-Cx-1 sample.

**Figure 2:**
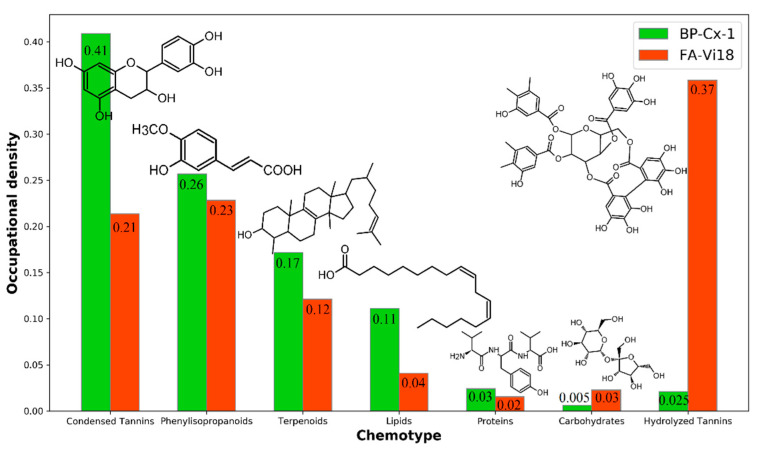
Occupational densities on van Krevelen diagram of each chemotype (condensed tannins, phenylisopropanoids, terpenoids, lipids, proteins, carbohydrates, hydrolyzed tannins) for BP-Cx-1 (green) and FA-Vi18 (orange), which were calculated according to Perminova [43].

Figure 2 visualizes the proportions of occupation densities (OD) in van Krevelen diagrams of general seven chemotypes characteristic for these two samples. These quantitative data confirm the qualitative observations outlined above. It can be seen that the BP-Cx-1 sample is enriched with the most hydrophobic and low oxidized molecular components, such as condensed tannins (OD 0.41 for BP-Cx-1 vs. OD 0.21 for FA-Vi18), phenylisopropanoids (0.26 vs. 0.23), and lipids (0.11 vs. 0.04), as compared to FA-Vi18. The most tangible difference is observed in the comparison of the population densities in the region of hydrolyzed tannins (O/C > 0.5, H/C < 1.4): FA-Vi18 is represented mainly by molecular components of this class, while there are no formulae of this chemotype in BP-Cx-1 (0.03 for BP-Cx-1 vs. 0.37 for FA-Vi18).

Thus, the conducted study on the molecular compositions of the BP-Cx-1 and FA-Vi18 samples allowed us to conclude that BP-Cx-1 is enriched with condensed aromatic hydrophobic components, whereas FA-Vi18 is rich in hydrophilic and oxidized molecular components of predominately hydrolyzed tannins chemotype.

**Figure 3:**
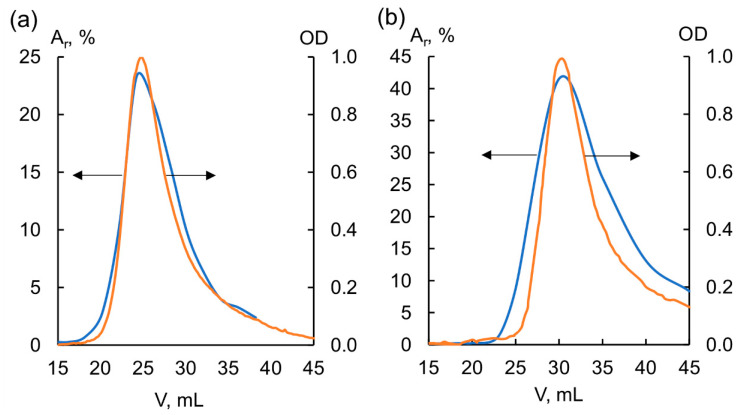
UV and radioactivity profiles of (**a**) tritium-labeled BP-Cx-1 and (**b**) tritium-labeled FA-Vi18. A_r_ is radioactivity normalized per total eluted radioactivity, OD is optical density. Radioactivity profile is highlighted in orange and its appropriate scale is indicated with a left-oriented-arrow, UV-profile is highlighted in blue, and its appropriate scale is indicated with a right-oriented arrow.

The following molecular weights were obtained for the analyzed samples: Mp = 5.9 kDa for FA-Vi18, and Mp = 4.7 kDa for BP-Cx-1. Due to the high hydrophobicity of BP-Cx-1, it was not possible to achieve its full recovery from the column. The molecular weight of peat fulvic acid correlates well with the data reported for peat HS [40]. In general, the FA sample can be characterized as predominantly aliphatic, highly oxidized, and with relatively high molecular weight. The lignin derivative, on the contrary, is characterized by high hydrophobicity, a low degree of oxidation, and a lower molecular weight. The radioactivity and UV profiles of both samples used for the subsequent studies are shown in Figure 3.

As seen in Figure 3, the radioactivity and UV profiles of both labeled samples are substantially similar. This confirms that the label was incorporated in all components of the molecular ensemble of the FA-Vi18 and BP-Cx-1 samples, which were used in subsequent bioavailability and tissue distribution studies. The suggested structures of the ^3^H-labeled components are shown in Supplementary Figure S1 (Supplementary Materials).

### 3.2. Distribution of the ^3^H-Labeled Fulvic Acid and BP Cx-1 in the Tissue of Mice

**Figure 4:**
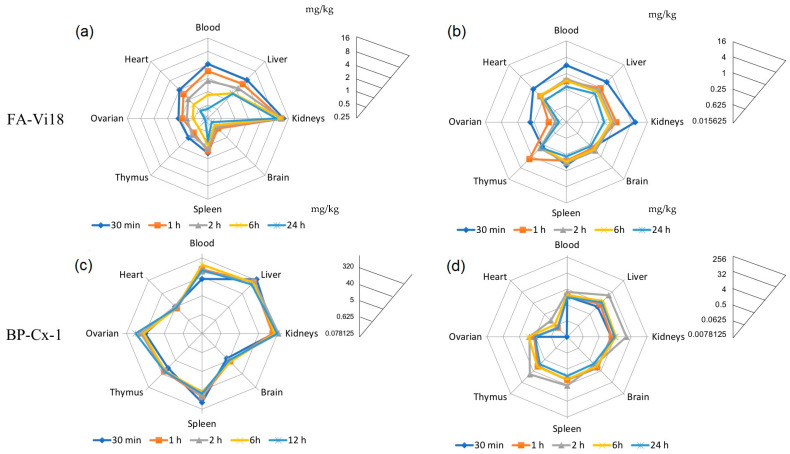
Distribution profiles resulting from Log2 scale transformation of the tissue concentrations of FA-Vi18 sample and the lignin derivative BP-Cx-1 administered to female BALB/c mice. The radar profiles of planes (**a**) and (**c**) correspond to single intravenous injection, and planes (**b**) and (**d**) correspond to single gavage administration of [^3^H]-labeled FA-Vi18 (10 mg/kg) and BP-Cx-1 (100 mg/kg), respectively.

The ^3^H-labeled samples of peat fulvic acids and of lignin derivative were administered intravenously and by gavage to mice. In the experiments with the ^3^H-labeled fulvic acids, none of the specific gross changes were observed in the internal organs of the sacrificed animals. Tritium-labeled compounds were detected in all analyzed organs and tissues, including the brain (Table 3). The tissue distribution profiles after intravenous and gavage administration are shown in Figure 4a,b. All concentrations of ^3^H-labeled compounds in the tissues were calculated using eq 1. C_max_ of [^3^H]-FA-Vi18 in all studied tissues was observed at 5 min after intravenous injection and was the highest in kidneys (28.53 mg/kg), blood (14.12 mg/kg), and liver (9.65 mg/kg), while the smallest values were observed in the brain (0.69 mg/kg) of mice.

The highest accumulation of [^3^H]-FA-Vi18 was observed in the kidneys (Figure 4a), reflected in the order of magnitude more significant value of AUC_0-24_ for this organ (250.86 h × mg × kg^−1^) and tissue-to-blood ratio (2.02), which may be related to the metabolism of FA-Vi18 in this organ. The largest half-lives were obtained for the liver (53.7 h), kidneys (52.2 h), and brain (48.8 h), while the smallest one was for the blood (11.7 h). Thus, when administered intravenously, FA was rapidly distributed from the bloodstream to the organs.

In the case of the oral administration, the Cmax in most tissues was reached in half an hour (blood, liver, kidneys, heart, spleen, ovary). The maximum concentration in the thymus was observed after 1 h, and in the brain after two hours. The highest concentrations were obtained in the kidneys (5.6 mg/kg), liver (2.02 mg/kg), and blood (1.99 mg/kg). The most considerable accumulation indicated by the AUC_0-48_ values of (25.32 h × mg × kg^−1^) and the values of tissue-to-blood ratio of 2.8 was observed in the kidneys after the intravenous injection (Table 4).

Almost identical AUC_0-inf_ for blood after oral and intravenous administration indicates a high absolute bioavailability of FA-Vi18 after oral administration. The half-life of [^3^H]-FA-Vi18 in the blood after oral administration (41 h) significantly exceeds that after intravenous administration (11.7 h). This, along with a high AUC_0-t_ value for blood, compared to organs, indicates the relatively slow distribution of the orally administered drug from the bloodstream (Figure 4b).

In the case of [^3^H]-BP-Cx-1, the brownish coloration of tissues and internal organs, except for the thymus, was observed in the necropsied animals intravenously administered with [^3^H]-Bp-Cx-1. Sixty minutes after intravenous injection of [^3^H]-BP-Cx-1, dark-brown staining of the contents of the small intestine and cecum was observed, most pronounced 12 h after the injection. This might suggest intestinal excretion of [^3^H]-Bp-Cx-1. No other gross changes of internal organs were observed. In mice necropsied 24 h after oral administration of [^3^H]-BP-Cx-1, it was observed inside the lumen of the ascending colon and in the cecum. No specific macroscopic changes of internal organs were noted.

The hydrophobic [^3^H]-BP-Cx-1 shows a slightly different pattern of organ distribution than [^3^H]-FA-Vi18 (Figure 4c,d). It showed approximately equal accumulation of the drug in the liver and kidneys with intravenous and oral administration. When administered intravenously, the value for the liver was 396.7 mg/kg (T_max_ = 5 min) and 343.3 mg/kg for the kidneys (T_max_ = 120 min), with accumulation coefficients of 2.56 and 2.21, respectively (Table 5). When administered orally, the drug displayed maximum concentration in these organs 120 min after injection. It was 16.13 mg/kg with an accumulation factor of 5.98 for the liver and 17.73 mg/kg with 6.57 for the kidneys (Table 6). These results indicate that [^3^H]-BP-Cx-1 is metabolized both by the liver and kidneys.

Compared to the fulvic acid, the lignin derivative has a higher affinity for ovarian tissues (tissue-to-blood ratio was 0.29–0.17 for [^3^H]-FA-Vi18 and 0.75–0.43 for [^3^H]-BP-Cx-1 with the intravenous and oral route introduction, respectively) and spleen (tissue-to-blood ratio 0.25–0.32 and 1.5–1.6 for the two drugs, respectively).

As in the case of [^3^H]-FA-Vi18, relatively low concentrations of [^3^H]-BP-Cx-1 were found in the heart (5.4 mg/kg with intravenous and 0.16 mg/kg for gavage administration) and in the brain (6.3 mg/kg). We also found a delayed dynamics of accumulation and excretion of [^3^H]-BP-Cx-1 from organs (Figure 4c,d): a significant decrease in concentration during the observation period with intravenous administration occurred only in the blood (half-life 6.8 h), liver (half-life 14.6 h), and kidneys (half-life 15.5 h) (Table 5). The elimination half-life of the gavage administered [^3^H]-BP-Cx-1 significantly exceeded that of the intravenous one and was the largest for the kidneys (226.8 h) (Table 6). AUC_0-inf_ for blood after gavage and intravenous administration accounted for 11.6%, indicating low bioavailability of gavage-administered [^3^H]-BP-Cx-1. The primary data on kinetics of tissue distribution of [^3^H]-FA-Vi18 and [^3^H]-BP-Cx-1 in mice are given in Supplementary Tables S1 and S2, respectively.

## 4. Discussion

The study was performed to compare the bioavailability and tissue distribution of two natural polyphenolic products: the polyphenolic ligand BP-Cx-1 (lignin derivative) and natural fulvic acids isolated from water-seepage of the underground peat deposit (FA-Vi18), administered intravenously or orally to Balb/C mice. The materials differed substantially in chemical structures and hydrophobicity: the lignin derivative was rich in hydrophobic low-oxidized structures such as condensed tannins, lignin-like molecules, and terpenoids, and completely lacked hydrolysable tannins, whereas the sample of fulvic acids was depleted of the least oxidized subgroups of condensed tannins and very rich in hydrolysable tannins. The observed differences in molecular compositions were in sync with the lower content of oxygen in the BP-Cx-1 sample as well as with the higher contribution of aromatic versus aliphatic carbon as determined from ^13^C NMR data. All structural parameters measured were indicative of much higher hydrophobicity of the lignin derivative used in this study (BP-Cx-1) as compared to the peat fulvic acid (FA-Vi18) sample. The obtained data corroborate well with the dominating types of structural motifs reported for lignins and fulvic acids, such as phenyisopropanoic units and hydrolyzable tannins, respectively [34,49].

For the purposes of this study, of particular importance is that despite the substantial differences in the partial structures of the phenolic compounds used in this study, application of the tritium bombardment technique gave consistent results for both materials. It was shown that the radioactivity and UV profiles of the labeled samples did not differ from the parent samples. This is indicative of homogeneous (non-selective) distribution of the label among all components of the molecular ensembles of the both polyphenolic samples used in this study, FA-Vi18 and BP-Cx-1.

We utilized the radioactive tritium label to detect the total concentration of the labeled samples and their metabolites in the blood and animals’ organs (the corresponding data are shown in Supplementary Tables S1 and S2). In our previous studies [38], we showed that the method of introduction of the tritium label does not affect the chemical properties of the labeled compounds and allowed us to surmise on the behavior of the parent compounds. To determine the range of concentrations, which can be reliably determined in vivo with the use of liquid scintillation technique after administration in the internal organs of mice, we used administration dosage covering an order of magnitude: 10 mg/kg in the case of FA-Vi18 sample and 100 mg/kg in the case of BP-Cx-1.

The present in vitro study showed that the maximum blood concentrations for the FA-Vi18 are at 14.12 mg/kg when administered intravenously and 1.99 mg/kg when gavage administered at a dose of 10 mg/kg. Moreover, when gavage administered, the liver, kidneys, and thymus accumulated the drug in high concentrations. In our previous studies on antiviral efficacy of humic materials of the similar nature, it was shown that the EC50 for HIV ranged from 0.98 to 6.7 mg/L [16]. At the same time, for the TBEV virus, the EC50 for the humic samples ranged from 0.14 mg/L to 0.9 mg/L [34]. In this study, for the polyphenolic BP-Cx-1, the maximum blood concentrations were 2.70 mg/kg when administered per gavage and 155.2 mg/kg when administered intravenously at a dose of 100 mg/kg. Thus, the present in vivo study showed that the previously reported effective doses for both drugs are achieved after a single administration including administration per gavage.

The proposed approach enabled quantitative comparison of the accumulation kinetics of the studied samples in various organs. The substantial differences were found between the hydrophilic fulvic acid and the hydrophobic lignin derivative. For the FA-Vi18 sample, regardless of the administration route, the kidneys were the organs of predominant retention, while the liver accumulated these compounds to a much lesser extent. BP-Cx-1 appeared to accumulate mainly in the liver and kidneys. Given that the tritium label remains in the metabolites of the tested compounds, we suggest that the kidneys and the liver are the major organs of metabolism of these compounds. Our conclusions seem to be in agreement with the data for other polyphenolic compounds, such as quercetin [50].

## 5. Conclusions

The use of tritium-labeled peat fulvic acids and lignin derivative approach appears to be well suited for studying the pharmacokinetics, absorption, distribution, metabolism, and excretion (ADME) of complex organic mixtures, such as humic substances (HS) and lignins. For complex multicomponent mixtures, the proposed method of non-selective tritium labeling with a use of the thermal bombardment technique has a substantial advantage over the traditional approaches for identification of individual chemical compounds in biological media, and it enables us to detect components of the labeled mixture regardless of their specific structural patterns. A ratio of aromatic to aliphatic moieties as measured by ^13^C NMR spectroscopy was shown to be a reliable predictor of lipophilicity of the polyphenolic mixtures in ADME studies. The obtained results may lay the groundwork for designing in vivo pharmacokinetic studies on complex polyphenolic drugs (e.g., HS) with the use of tritium label. The proposed approach can be easily extended to other complex natural products, their metabolites, and phytochemicals, affirming its broad scientific significance.

## Figures and Tables

**Table 1:** Elemental composition of the fulvic acid and of the lignin-derived substance samples under study (on an ash-free basis).

Sample	C%	H%	N%	O%	O/C	H/C
Fulvic acid (FA-Vi18)	49.14	5.19	1.29	44.38	0.68	1.27
Lignin derivative (BP-Cx-1)	65.60	4.11	0.00	30.29	0.35	0.75

**Table 2:** Distribution of carbon among the major structural groups in the studied fulvic acid and BP-Cx-1 samples (% of total C atoms by the ^13^C NMR data).

Sample	CH_n_	CH_3_O	CalkO	Car	CarO	COO	C = O	ΣCar	ΣCar/ΣCalk
FA-Vi18	28	5	12	22	8	20	5	30	0.67
BP-Cx-1	20	5	11	39	8	11	7	47	1.3

**Table 3:** The main pharmacokinetic parameters after intravenous injection of [^3^H]-FA-Vi18 at 10 mg/kg to female BALB/c mice (*n* = 3).

Index	Organ or Tissue							
	Blood	Liver	Kidneys	Brain	Spleen	Thymus	Ovaries	Heart
C_max_, mg/kg	14.12	9.65	28.53	0.69	3.60	2.57	4.14	6.06
Tissue-to-blood ratio	1.00	0.68	2.02	0.05	0.25	0.18	0.29	0.43
T_max_, min	5	5	5	5	5	5	5	5
AUC_0-24_ h × mg × kg^−1^	27.79	45.08	250.86	9.36	23.94	12.07	13.62	18.75
Lz, min^−1^ × kg^−1^	0.059	0.013	0.013	0.014	0.016	0.040	0.038	0.036
AUC_0-inf_, h × mg × kg^−1^	34.6	164.2	884.9	31.9	71.8	19.6	21.6	31.2
T_1/2_, h	11.7	53.7	52.2	48.0	42.0	17.4	18.4	19.7
MRT, h	12.6	76.4	74.1	69.0	59.7	24.0	23.6	25.7

**Table 4:** The main pharmacokinetic parameters after gavage administration of [^3^H]-FA-Vi18 at 10 mg/kg to female BALB/c mice (*n* = 3).

Index	Organ or Tissue							
	Blood	Liver	Kidneys	Brain	Spleen	Thymus	Ovaries	Heart
C_max_, mg/kg	1.99	2.02	5.6	0.51	0.64	1.37	0.34	0.86
Tissue-to-blood ratio	1.00	1.01	2.8	0.25	0.32	0.69	0.17	0.43
T_max_, min	30	30	30	120	30	60	30	30
AUC_0-48_ h × mg × kg^−1^	18.15	24.11	25.32	15.46	16.84	13.13	1.72	789
Lz, h^−1^ × kg^−1^	0.017	0.015	0.014	0.011	0.010	0.007	-	0.0001
AUC_0-inf_, h × mg ×kg^−1^	33.3	45.2	54.0	38.2	45.4	48.6	-	47.7
T_1/2_, h	41.0	44.9	50.9	60.6	66.9	96.8	-	88.7
MRT, h	59.3	63.7	73.4	89.1	98.8	142.9	-	133.5

**Table 5:** The main pharmacokinetic parameters after intravenous injection of [^3^H]-BP-Cx-1 at 100 mg/kg to female BALB/c mice (*n* = 3).

Index	Organ or Tissue							
	Blood	Liver	Kidneys	Brain	Spleen	Thymus	Ovaries	Heart
C_max_, mg/kg	155.2	396.7	343.3	6.3	233.3	28.2	115.7	5.4
Tissue-to-blood ratio	1.00	2.56	2.21	0.04	1.50	0.18	0.75	0.03
T_max_, min	360	5	120	360	5	60	720	5
AUC_0-12_ h × mg × kg^−1^	1297.6	2650.0	3134.2	64.9	756.6	300.4	808.2	53.0
Lz, h^−1^ × kg^−1^	0.102	0.047	-	0.045	0.063	-	-	-
AUC_0-inf_, h × mg × kg^−1^	2138.3	6242.5	-	173.9	1649.0	-	-	-
T_1/2_, h	6.8	14.6	-	15.5	10.9	-	-	-
MRT, h	15.5	-	-	64.7	11.5	-	-	-

**Table 6:** The main pharmacokinetic parameters after gavage administration of [^3^H]-BP-Cx-1 at 100 mg/kg to female BALB/c mice (*n* = 3).

Index	Organ or Tissue							
	Blood	Liver	Kidneys	Brain	Spleen	Thymus	Ovaries	Heart
C_max_, mg/kg	2.70	16.13	17.73	2.10	4.33	7.33	1.17	0.16
Tissue-to-blood ratio	1.00	5.98	6.57	0.78	1.60	2.72	0.43	0.06
T_max_, min	120	120	120	30	120	120	360	120
AUC_0-48_ h × mg × kg^−1^	73.1	275.4	227.7	58.3	74.7	76.0	36.4	2.9
Lz, h^−1^ × kg^−1^	0.007	-	0.003	0.012	0.010	0.015	-	0.005
AUC_0-inf_, h × mg × kg^−1^	248.5	-	1536.6	136.4	193.5	135.4	-	15.9
T_1/2_, h	91.2	-	226.8	55.9	68.6	44.1	-	150.5
MRT, h	133.1	-	322.6	82.6	97.8	58.4	-	221.1

## Data Availability

Data supporting the obtained results can be found in the Supplementary materials to this manuscript.

## Supplementary Materials

The following are available online at https://www.mdpi.com/article/10.3390/biomedicines9121787/s1, Figure S1: Schematic representation of the structure of the labeled products within FA or lignin derivative mixtures after application of thermal tritium bombardment technique (left side structure) followed by purification from the mobile ^3^H atoms with a use of dialysis against 0.028 M phosphate buffer (right side structure). The resulting products carry only immobile ^3^H atoms within skeleton structures of FA and lignin derivative, Table S1: Tissue content of [^3^H]-FA-Vi18 after intravenous injection or by gavage of 10 mg/kg to female BALB/C mice (M ± SEM, *n* = 3), Table S2: Tissue content of [^3^H]-BP-Cx-1 after intravenous injection or by gavage of 100 mg/kg to female BALB/C mice (M ± SEM, *n* = 3). Supplementary materials contain Figure S1, which shows dialysis process and feasible structures of the resulting labeled products, Tables S1 and S2 present the data on tissue and organs distribution of [^3^H]-FA-Vi18 and [^3^H]-BP-Cx-1 in mice, respectively.

## References

[1] Bent S. (2008). Herbal Medicine in the United States: Review of Efficacy, Safety, and Regulation—Grand Rounds at University of California, San Francisco Medical Center. J. Gen. Intern. Med..

[2] Sun S., Wang Y., Wu A., Ding Z., Liu X. (2019). Influence Factors of the Pharmacokinetics of Herbal Resourced Compounds in Clinical Practice. Evid.-Based Complement. Altern. Med..

[3] Murakami A. (2014). Dose-Dependent Functionality and Toxicity of Green Tea Polyphenols in Experimental Rodents. Arch. Biochem. Biophys..

[4] D’Angelo S., Martino E., Ilisso C.P., Bagarolo M.L., Porcelli M., Cacciapuoti G. (2017). Pro-Oxidant and pro-Apoptotic Activity of Polyphenol Extract from Annurca Apple and Its Underlying Mechanisms in Human Breast Cancer Cells. Int. J. Oncol..

[5] EMEA (2000). Products Guidelines for the Conduct of Pharmacokinetic Studies in Target Animal Species (EMEA/CVMP/133/99-FINAL).

[6] Espinoza-Acosta J.L., Torres-Chávez P.I., Ramírez-Wong B., López-Saiz C.M., Montaño-Leyva B. (2016). Antioxidant, Antimicrobial, and Antimutagenic Properties of Technical Lignins and Their Applications. BioResources.

[7] Ugartondo V., Mitjans M., Vinardell M.P. (2008). Comparative Antioxidant and Cytotoxic Effects of Lignins from Different Sources. Bioresour. Technol..

[8] Fedoros E.I., Orlov A.A., Zherebker A., Gubareva E.A., Maydin M.A., Konstantinov A.I., Krasnov K.A., Karapetian R.N., Izotova E.I., Pigarev S.E. (2018). Novel Water-Soluble Lignin Derivative BP-Cx-1: Identification of Components and Screening of Potential Targets in Silico and in Vitro. Oncotarget.

[9] Anisimov V.N., Popovich I.G., Zabezhinski M.A., Yurova M.N., Tyndyk M.L., Anikin I.V., Egormin P.A., Baldueva I.A., Fedoros E.I., Pigarev S.E. (2016). Polyphenolic Drug Composition Based on Benzenepolycarboxylic Acids (BP-C3) Increases Life Span and Inhibits Spontaneous Tumorigenesis in Female SHR Mice. Aging.

[10] Panchenko A.V., Fedoros E.I., Pigarev S.E., Maydin M.A., Gubareva E.A., Yurova M.N., Kireeva G.S., Lanskikh G.P., Tyndyk M.L., Anisimov V.N. (2018). Effect of the Polyphenol Composition BP-C3 on Haematological and Intestinal Indicators of 5-Fluorouracil Toxicity in Mice. Exp. Ther. Med..

[11] Bykov V.N., Drachev I.S., Kraev S.Y., Maydin M.A., Gubareva E.A., Pigarev S.E., Anisimov V.N., Baldueva I.A., Fedoros E.I., Panchenko A. (2018). V Radioprotective and Radiomitigative Effects of BP-C2, a Novel Lignin-Derived Polyphenolic Composition with Ammonium Molybdate, in Two Mouse Strains Exposed to Total Body Irradiation. Int. J. Radiat. Biol..

[12] Aeschbacher M., Graf C., Schwarzenbach R.P., Sander M. (2012). Antioxidant Properties of Humic Substances. Environ. Sci. Technol..

[13] Perminova I.V., Kovalenko A.N., Schmitt-Kopplin P., Hatfield K., Hertkorn N., Belyaeva E.Y., Petrosyan V.S. (2005). Design of Quinonoid-Enriched Humic Materials with Enhanced Redox Properties. Environ. Sci. Technol..

[14] Klein O.I., Kulikova N.A., Filimonov I.S., Koroleva O.V., Konstantinov A.I. (2018). Long-Term Kinetics Study and Quantitative Characterization of the Antioxidant Capacities of Humic and Humic-like Substances. J. Soils Sediment..

[15] Tarasova A.S., Stom D.I., Kudryasheva N.S. (2015). Antioxidant Activity of Humic Substances via Bioluminescent Monitoring in Vitro. Environ. Monit. Assess..

[16] Zhernov Y.V., Kremb S., Helfer M., Schindler M., Harir M., Mueller C., Hertkorn N., Avvakumova N.P., Konstantinov A.I., Brack-Werner R. (2016). Supramolecular Combinations of Humic Polyanions as Potent Microbicides with Polymodal Anti-HIV-Activities. New J. Chem..

[17] Van Rensburg C.E.J. (2015). The Antiinflammatory Properties of Humic Substances: A Mini Review. Phytother. Res..

[18] Mirza M.A., Ahmad N., Agarwal S.P., Mahmood D., Anwer M.K., Iqbal Z. (2011). Comparative Evaluation of Humic Substances in Oral Drug Delivery. Results Pharma Sci..

[19] Mirza M.A. (2018). Future of Humic Substances as Pharmaceutical Excipient. Pharm. Sci. Anal. Res. J..

[20] Zykova M.V., Schepetkin I.A., Belousov M.V., Krivoshchekov S.V., Logvinova L.A., Bratishko K.A., Yusubov M.S., Romanenko S.V., Quinn M.T. (2018). Physicochemical Characterization and Antioxidant Activity of Humic Acids Isolated from Peat of Various Origins. Molecules.

[21] Winkler J., Ghosh S. (2018). Therapeutic Potential of Fulvic Acid in Chronic Inflammatory Diseases and Diabetes. J. Diabetes Res..

[22] Kala K.J., Prashob P.K.J., Chandramohanakumar N. (2019). Humic Substances as a Potent Biomaterials for Therapeutic and Drug Delivery System-a Review. Int. J. Appl. Pharm..

[23] Fan X., Xu Y., Zhu D., Ji Y. (2017). Pharmacokinetic Comparison of 20(R)- and 20(S)-Ginsenoside Rh1 and 20(R)- and 20(S)-Ginsenoside Rg3 in Rat Plasma Following Oral Administration of Radix Ginseng Rubra and Sheng-Mai-San Extracts. Evid.-Based Complement. Altern. Med. eCAM.

[24] Wang H.C., Bao Y.R., Wang S., Li T.J., Meng X.S. (2019). Simultaneous Determination of Eight Bioactive Components of Cirsium Setosum Flavonoids in Rat Plasma Using Triple Quadrupole LC/MS and Its Application to a Pharmacokinetic Study. Biomed. Chromatogr..

[25] Guo Z., Lou Y., Kong M., Luo Q., Liu Z., Wu J. (2019). A Systematic Review of Phytochemistry, Pharmacology and Pharmacokinetics on Astragali Radix: Implications for Astragali Radix as a Personalized Medicine. Int. J. Mol. Sci..

[26] Hertkorn N., Harir M., Koch B.P., Michalke B., Schmitt-Kopplin P. (2013). High-field NMR Spectroscopy and FTICR Mass Spectrometry: Powerful Discovery Tools for The Molecular Level Characterization of Marine Dissolved Organic Matter. Biogeosciences.

[27] Roullier-Gall C., Signoret Julie H.D., Witting M.A., Kanawati B., Schäfer B., Gougeon R.D., Schmitt-Kopplin P. (2018). Usage of FT-ICR-MS Metabolomics for Characterizing the Chemical Signatures of Barrel-Aged Whisky. Front. Chem..

[28] Kim C.H., Kim M.Y., Lee S.W., Jang K.-S. (2019). UPLC/FT-ICR MS-Based High-Resolution Platform for Determining the Geographical Origins of Raw Propolis Samples. J. Anal. Sci. Technol..

[29] Bowman A.P., Blakney G.T., Hendrickson C.L., Ellis S.R., Heeren R.M.A., Smith D.F. (2020). Ultra-High Mass Resolving Power, Mass Accuracy, and Dynamic Range MALDI Mass Spectrometry Imaging by 21-T FT-ICR MS. Anal. Chem..

[30] Derrien M., Lee Y.K., Park J.E., Li P., Chen M., Lee S.H., Lee S.H., Lee J.B., Hur J. (2017). Spectroscopic and Molecular Characterization of Humic Substances (HS) from Soils and Sediments in a Watershed: Comparative Study of HS Chemical Fractions and the Origins. Environ. Sci. Pollut. Res..

[31] Ware S.A., Hartman B.E., Waggoner D.C., Vaughn D.R., Bianchi T.S., Hatcher P.G. (2021). Molecular Evidence for the Export of Terrigenous Organic Matter to the North Gulf of Mexico by Solid-State ^13^C NMR and Fourier Transform Ion Cyclotron Resonance Mass Spectrometry of Humic Acids. Geochim. Cosmochim. Acta.

[32] D’Auria M., Emanuele L., Racioppi R. (2011). A FT-ICR MS Study of Lignin: Determination of An Unusual Structural Repetitive Unit in Steam Exploded Lignin From Wheat Straw. Lett. Org. Chem..

[33] Qi Y., Fu P., Volmer D. (2020). Analysis of Natural Organic Matter via Fourier Transform Ion Cyclotron Resonance Mass Spectrometry: An Overview of Recent Non-Petroleum Applications. Mass Spectrom. Rev..

[34] Orlov A.A., Zherebker A., Eletskaya A.A., Chernikov V.S., Kozlovskaya L.I., Zhernov Y.V., Kostyukevich Y., Palyulin V.A., Nikolaev E.N., Osolodkin D.S. (2019). Examination of molecular space and feasible structures of bioactive components of humic substances by FTICR MS data mining in ChEMBL database. Sci. Rep..

[35] Leyva D., Tose L.V., Porter J., Wolff J., Jaffé R., Fernandez-Lima F. (2019). Understanding the Structural Complexity of Dissolved Organic Matter: Isomeric Diversity. Faraday Discuss..

[36] Badun G.A., Chernysheva M.G., Tyasto Z.A., Kulikova N.A., Kudryavtsev A.V., Perminova I.V. (2010). A New Technique for Tritium Labeling of Humic Substances. Radiochim. Acta.

[37] Kulikova N.A., Perminova I.V., Badun G.A., Chernysheva M.G., Koroleva O.V., Tsvetkova E.A. (2010). Estimation of Uptake of Humic Substances from Different Sources by Escherichia Coli Cells under Optimum and Salt Stress Conditions by Use of Tritium-Labeled Humic Materials. Appl. Environ. Microbiol..

[38] Kulikova N.A., Abroskin D.P., Badun G.A., Chernysheva M.G., Korobkov V.I., Beer A.S., Tsvetkova E.A., Senik S.V., Klein O.I., Perminova I.V. (2016). Label Distribution in Tissues of Wheat Seedlings Cultivated with Tritium-Labeled Leonardite Humic Acid. Sci. Rep..

[39] Hertkorn N., Permin A., Perminova I., Kovalevskii D., Yudov M., Petrosyan V., Kettrup A. (2002). Comparative Analysis of Partial Structures of a Peat Humic and Fulvic Acid Using One- and Two-Dimensional Nuclear Magnetic Resonance Spectroscopy. J. Environ. Qual..

[40] Perminova I.V., Frimmel F.H., Kudryavtsev A.V., Kulikova N.A., Abbt-Braun G., Hesse S., Petrosyan V.S. (2003). Molecular Weight Characteristics of Humic Substances from Different Environments as Determined by Size Exclusion Chromatography and Their Statistical Evaluation. Environ. Sci. Technol..

[41] Mueller C., Kremb S., Gonsior M., Brack-Werner R., Voolstra C.R., Schmitt-Kopplin P. (2020). Advanced identification of global bioactivity hotspots via screening of the metabolic fingerprint of entire ecosystems. Sci. Rep..

[42] Koch B.P., Dittmar T., Witt M., Kattner G. (2007). Fundamentals of Molecular Formula Assignment to Ultrahigh Resolution Mass Data of Natural Organic Matter. Anal. Chem..

[43] Perminova I.V. (2019). From Green Chemistry and Nature-like Technologies towards Ecoadaptive Chemistry and Technology. Pure Appl. Chem..

[44] Badun G.A., Chernysheva M.G., Ksenofontov A.L. (2012). Increase in the Specific Radioactivity of Tritium-Labeled Compounds Obtained by Tritium Thermal Activation Method. Radiochim. Acta.

[45] Perminova I.V., Frimmel F.H., Kovalevskii D.V., Abbt-Braun G., Kudryavtsev A.V., Hesse S. (1998). Development of a Predictive Model for Calculation of Molecular Weight of Humic Substances. Water Res..

[46] Li J., Yan K., Hou L., Du X., Zhu P., Zheng L., Zhu C. (2017). An Algorithm and R Program for Fitting and Simulation of Pharmacokinetic and Pharmacodynamic Data. Eur. J. Drug Metab. Pharmacokinet..

[47] Rice J.A., MacCarthy P. (1991). Statistical Evaluation of the Elemental Composition of Humic Substances. Org. Geochem..

[48] Perminova I.V., Grechishcheva N.Y., Petrosyan V.S. (1999). Relationships between Structure and Binding Affinity of Humic Substances for Polycyclic Aromatic Hydrocarbons: Relevance of Molecular Descriptors. Environ. Sci. Technol..

[49] Sakakibara A. (1980). A Structural Model of Softwood Lignin. Wood Sci. Technol..

[50] Wang W., Sun C., Mao L., Ma P., Liu F., Yang J., Gao Y. (2016). The Biological Activities, Chemical Stability, Metabolism and Delivery Systems of Quercetin: A Review. Trends Food Sci. Technol..

