# Supplementary material for: A Use of Tritium-Labeled Peat Fulvic Acids and Polyphenolic Derivatives for Designing Pharmacokinetic Experiments on Mice
Source: Biomedicines. 2021 Nov 29;9(12):1787. doi: 10.3390/biomedicines9121787 (PMC8698565; doi:10.3390/biomedicines9121787)
Supplement: Supplementary file 1 [file biomedicines-09-01787-s001.zip › biomedicines-1466482-SI.pdf]

Supplementary Materials

Table S1. Tissue content of [^{3}H]-FA-Vi18 after intravenous injection or by gavage of 10 mg/kg to female BALB/C mice (M±SEM, n=3)

A. Tissue content after intravenous injection (mg/kg)						
Sample	Time after administration					
	5 min	30 min	1 h	2 h	6 h	24 h
Blood	14,12±1,34	4,21±1,44	2,91±0,48	1,78±0,77	0,85±0,72	0,41±0,38
Liver	9,65±0,96	4,26±0,28	3,15±0,36	2,29±0,12	1,56±0,10	1,54±0,07
Kidneys	28,53±1,34	12,28±1,44	10,67±0,48	11,05±0,77	11,15±0,72	8,43±0,38
Brain	0,69±0,03	0,51±0,02	0,52±0,04	0,46±0,02	0,41±0,03	0,33±0,01
Spleen	3,60±0,22	1,49±0,17	1,34±0,10	1,19±0,05	0,97±0,10	0,79±0,06
Thymus	2,57±0,17	1,02±0,02	0,70±0,04	0,81±0,09	0,49±0,03	0,30±0,04
Ovaries	4,14±1,34	1,14±1,44	0,94±0,48	0,73±0,77	0,54±0,72	0,30±0,38
Heart	6,06±0,38	2,00±0,17	1,49±0,11	1,04±0,03	0,70±0,05	0,44±0,02
B. Tissue content after oral administration (mg/kg)						
Sample	Time after administration					
	30 min	1 h	2 h	6 h	24 h	48 h
Blood	1,99±4,67	0,51±0,09	0,61±0,07	0,53±0,09	0,32±0,01	0,26±0,05
Liver	2,02±1,29	0,94±0,11	0,77±0,02	0,62±0,06	0,49±0,14	0,32±0,03
Kidneys	5,60±4,67	1,11±0,09	0,85±0,07	0,71±0,09	0,40±0,01	0,39±0,05
Brain	0,35±0,08	0,37±0,04	0,51±0,05	0,43±0,02	0,29±0,01	0,26±0,01
Spleen	0,64±0,36	0,43±0,05	0,51±0,02	0,47±0,03	0,30±0,01	0,30±0,04
Thymus	0,30±0,04	1,37±1,04	0,38±0,03	0,34±0,04	0,35±0,02	0,22±0,01
Ovaries	0,34±4,67	0,07±0,09	0,04±0,07	0,03±0,09	0,03±0,01	0,05±0,05
Heart	0,86±0,58	0,36±0,01	0,36±0,05	0,39±0,02	0,21±0,02	0,27±0,04

Table S2. Tissue content of [^{3}H]-BP-Cx-1 after intravenous injection or by gavage of 100 mg/kg to female BALB/C mice (M±SEM, n=3)

A. Tissue content after intravenous injection (mg/kg)						
Sample	Time after administration					
	5 min	30 min	1 h	2 h	6 h	12 h
Blood	25,6±5,9	32,3±2,9	91,3±5,8	72,5±2,9	155,2±16,7	85,9±17,8
Liver	396,7±26,0	366,7±17,6	230,0±60,3	253,3±31,8	220,0±25,2	170,0±11,6
Kidneys	233,3±29,6	206,7±8,8	180,0±100,2	343,3±98,2	226,7±33,3	293,3±32,83
Brain	3,9±0,4	3,7±0,0	5,9±0,4	4,9±0,3	6,3±0,5	4,9±0,50
Spleen	233,3±52,4	153,3±8,8	80,0±25,2	73,3±8,8	46,7±8,8	56,7±8,8
Thymus	23,5±2,7	16,1±0,8	28,2±1,1	23,9±3,6	25,6±1,6	27,2±6,7
Ovaries	42,0±1,7	40,7±4,7	63,0±1,2	63,7±0,7	47,7±4,8	115,7±26,7
Heart	5,4±0,4	5,3±1,2	3,9±0,8	5,3±0,4	4,2±0,7	4,2±0,9
B. Tissue content after oral administration (mg/kg)						
Sample	Time after administration					
	30 min	1 h	2 h	6 h	24 h	48 h
Blood	1,50±0,10	1,63±0,26	2,70±1,86	1,87±0,17	1,33±0,41	1,33±0,34
Liver	2,10±0,17	3,37±0,52	16,13±12,87	5,80±0,20	4,67±1,39	5,73±1,82
Kidneys	2,50±0,15	2,53±0,22	17,73±15,39	4,60±0,46	3,83±0,73	4,00±1,08
Brain	2,10±0,10	1,93±0,24	1,30±0,32	1,73±0,23	1,07±0,26	0,97±0,22
Spleen	1,97±0,12	2,00±0,21	4,33±3,19	1,87±0,24	1,27±0,32	1,20±0,35
Thymus	1,73±0,19	1,77±0,26	7,33±6,39	1,83±0,29	1,13±0,27	0,93±0,28
Ovaries	0,47±0,32	0,58±0,19	1,03±0,83	1,17±0,12	0,53±0,23	0,77±0,22
Heart	0,008±0,002	0,045±0,025	0,160±0,145	0,077±0,027	0,041±0,023	0,060±0,023
