# Supplementary figures and images for: A Use of Tritium-Labeled Peat Fulvic Acids and Polyphenolic Derivatives for Designing Pharmacokinetic Experiments on Mice
Source: Biomedicines. 2021 Nov 29;9(12):1787. doi: 10.3390/biomedicines9121787 (PMC8698565; doi:10.3390/biomedicines9121787)

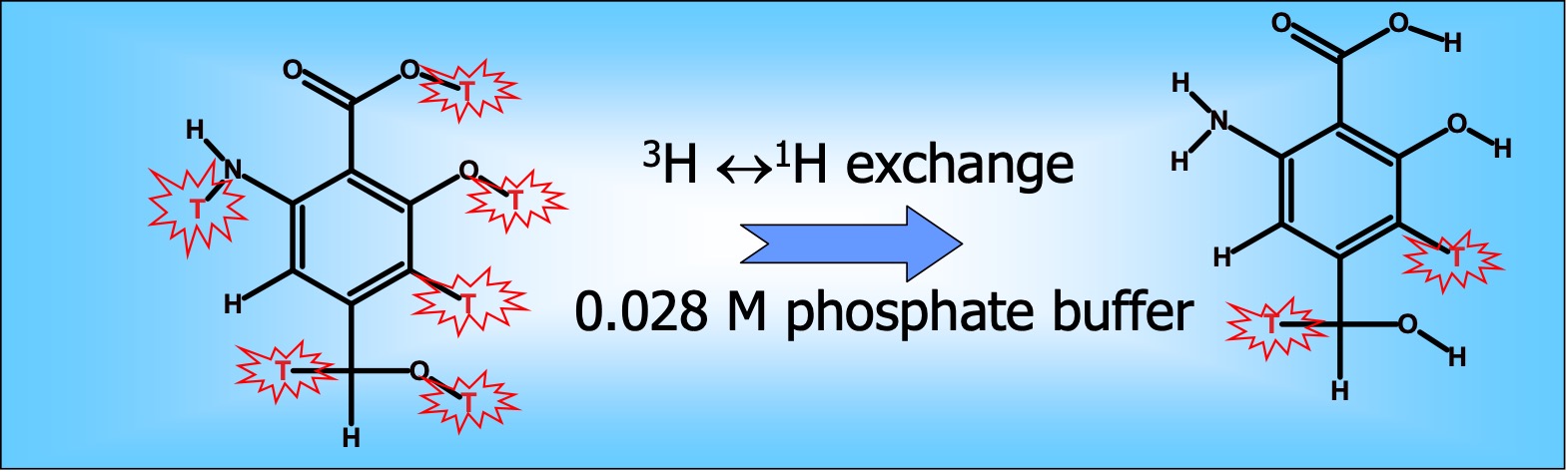

Supplement: Supplementary file 1 [file biomedicines-09-01787-s001.zip › biomedicines-1466482-Figure S1.jpg]
